# Bio-efficacy of Olyset^®^ Plus, PermaNet^®^ 3.0 and Interceptor^®^ G2 on pyrethroid-resistant populations of *Anopheles gambiae* s.l. prior to the June 2023 net distribution campaign in Benin, West Africa

**DOI:** 10.1186/s41182-024-00599-z

**Published:** 2024-04-30

**Authors:** David Mahouton Zoungbédji, Germain Gil Padonou, Arthur Sovi, Alphonse Keller Konkon, Albert Sourou Salako, Roseric Azondékon, Aboubakar Sidick, Juvénal Minassou Ahouandjinou, Linda Towakinou, Razaki Ossè, Rock Aïkpon, Cyriaque Affoukou, Lamine Baba-Moussa, Martin Akogbéto

**Affiliations:** 1 Centre de Recherche Entomologique de Cotonou (CREC), 06 BP 2604, Cotonou, Benin; 2 Faculté des Sciences et Techniques de l’Université d’Abomey-Calavi, Abomey-Calavi, Benin; 3 Programme National de Lutte Contre le Paludisme, Cotonou, Benin; 4 École de Gestion et d’exploitation des Systèmes d’élevage, Université Nationale d’Agriculture, Kétou, Benin; 5 Faculty of Agronomy, University of Parakou, Parakou, Benin; 6 Faculty of Infectious and Tropical Diseases, The London School of Hygiene and Tropical Medicine, London, UK; 7 Université Nationale des Sciences, Technologies, Ingénierie et Mathématiques (UNSTIM), Abomey, Benin; 8 Laboratoire de Biologie et de Typage Moléculaire en Microbiologie (LBTMM), département de Biochimie et de Biologie Cellulaire (BBC), Université de Abomey-Calavi (UAC), Abomey-Calavi, Benin

**Keywords:** *An. gambiae* s.l., Resistance, Insecticides, LLINs, Benin

## Abstract

**Background:**

This study investigates the effectiveness of new-generation mosquito nets, like Olyset^®^ Plus and PermaNet^®^ 3.0, and dual-action nets such as Interceptor^®^ G2, against pyrethroid-resistant *Anopheles gambiae* mosquitoes following the 2023 mass distribution of long-lasting insecticidal nets in Benin.

**Methods:**

We tested wild mosquito populations from six communes in Benin against various pyrethroid (permethrin 0.75%, alphacypermethrin 0.05%, and deltamethrin 0.05%) using WHO tube tests. Additionally, we exposed mosquitoes to chlorfenapyr 100 µg/ml using the CDC bottle bioassay method. A subset of mosquitoes underwent biochemical and PCR tests to check the overexpression of metabolic enzymes and the Kdr L1014F mutation. We evaluated the effectiveness of Olyset^®^ Plus, PermaNet^®^ 3.0, and Interceptor^®^ G2 nets using cone and tunnel tests on both laboratory and field populations of *An. gambiae*.

**Results:**

Overall, the highest mortality rate was 60% with pyrethroid and 98 to100% with chlorfenapyr. In cone tests, all three types of nets induced mortality rates above 80% in the susceptible laboratory strain of *An. gambiae*. Notably, Olyset^®^ Plus showed the highest mortality rates for pyrethroid-resistant mosquitoes in cone tests, ranging from 81.03% (95% CI: 68.59–90.13) in Djougou to 96.08% (95% CI: 86.54–99.52) in Akpro-Missérété. PermaNet^®^ 3.0 had variable rates, from 42.5% (95% CI: 27.04–59.11) in Djougou to 58.54% (95% CI: 42.11–73.68) in Porto-Novo. However, revealed good results for Interceptor^®^ G2, with 94% (95% CI: 87.40–97.77) mortality and 89.09% blood sampling inhibition in local populations of *An. gambiae*. In comparison, Interceptor^®^ had lower rates of 17% (95% CI: 10.23–25.82) and 60%, respectively.

**Conclusion:**

These results suggest that tunnel tests are effective for evaluating dual-active ingredient nets. Additionally, Interceptor^®^ G2 and PBO nets like Olyset^®^ Plus could be considered as alternatives against pyrethroid-resistant mosquitoes.

## Introduction

Indoor residual spraying (IRS) and long-lasting insecticidal nets (LLINs) are the two primary vector control tools recommended by the World Health Organization (WHO) for the management of malaria vectors. LLINs are extensively used in Africa as a preventive measure against malaria infection [1]. In 2021, the WHO estimated that 68% of the population in sub-Saharan Africa had access to at least one LLIN, marking a substantial increase from only 2% in 2000 [2]. This widespread use of LLINs has significantly reduced the burden of malaria in many sub-Saharan African countries [3, 4]. Notably, 69% of the 663 million cases of malaria averted in sub-Saharan Africa between 2001 and 2015 were attributed to LLINs [5].

Despite these positive outcomes, the rise of pyrethroid resistance, particularly in Benin, poses a serious threat to sustaining these achievements [6, 7]. Studies conducted in Benin attribute insecticide resistance to the overexpression of detoxification enzymes [8, 9], and mutations in specific genes, including *kdr* L1014F, *kdr* N1575Y, and Ace-1 G119S, found in populations of *An. gambiae* s.l. [10–12]. Given the context of multiple resistance mechanisms, there is a need to explore new tools for improved vector control.

An early response to insecticide resistance was the development of LLINs incorporating a pyrethroid and a synergist, piperonyl butoxide (PBO), designed to restore pyrethroid toxicity in resistant mosquitoes by inhibiting cytochrome P450 mono-oxygenase enzymes [13]. Two types of these LLINs, namely Olyset^®^ Plus and PermaNet^®^ 3.0, have been developed and prequalified by the WHO. Randomized controlled trials have demonstrated the superior performance of LLINs combining a pyrethroid and PBO compared to standard LLINs in Tanzania, Togo, and Burkina Faso [14, 15]. However, the effectiveness of these ITNs in a given area depends on the extent to which mono-oxygenase enzymes are involved in vector populations. In Benin, phase I evaluations revealed PermaNet^®^ 3.0LLINs induced mortalities exceeding 75% in local populations of Anopheles mosquitoes carrying several resistance mechanisms (*kdr* + detoxification enzymes) [16]. However, in experimental cases, Ngofur et al. [17] showed that Olyset^®^ Plus outperformed PermaNet^®^ 3.0in improving mosquito mortality compared to a standard pyrethroid-based net after multiple standardized washings.

To address target-modifying resistance observed with pyrethroids, duel-active impregnated nets were developed, featuring both a pyrethroid and an insecticide with a different mode of action, such as pyriproxyfen (PPF) or chlorfenapyr. PPF acts as an insect growth regulator, interfering with the metamorphosis of mosquito imaginal stages, while chlorfenapyr disrupts oxidative phosphorylation in insect mitochondria [18–20]. Recently, LLINs incorporating both alphacypermethrin and chlorfenapyr, known as Interceptor G2, were recommended by the WHO following successful randomized controlled trials in Tanzania [21] and Benin [22].

In Benin, the introduction of new-generation nets (Olyset^®^ Plus: PBO + permethrin, PermaNet^®^ 3.0: PBO + deltamethrin, and Interceptor^®^ G2 LLINs: chlorfenapyr + alphacypermethrin) in June 2023, represents a potential solution for overcoming and managing vector resistance. However, a previous study indicated that the addition of the PBO synergist did not fully restore vector susceptibility to pyrethroids [23]. However, a previous study indicated that the addition of the PBO synergist did not fully restore vector susceptibility to pyrethroids [23], even as the country plans to deploy PBO LLINs. Additionally, very few Phase I studies have assessed the bioefficacy of dual active-ingredient LLINs like Interceptor G2 on vector populations in Benin's various agro-ecological zones.

The current study undertaken in April 2023 aims to provide crucial information on the efficacy of brand new mosquito nets of which PBO LLIN and Interceptor G2 against different populations of *An. gambiae* s.l. in Phase I. This data aims to assist Benin National Malaria Control Program in making informed decisions regarding the selection of beneficiary areas for the different types of nets.

## Materials and methods

### Study area

**Fig. 1:**
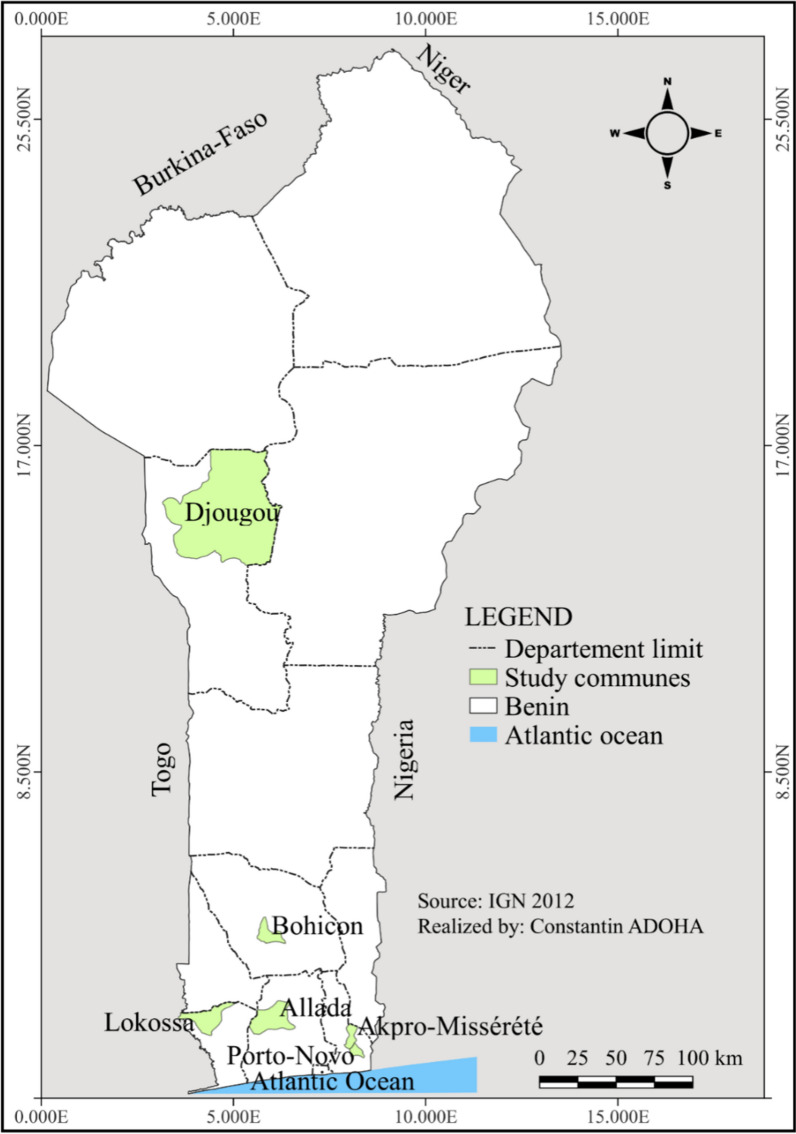
Study area

The study was carried out in the departments of Ouémé (communes of Akpro-Missérété and Porto-Novo), Atlantique (commune of Allada), Zou (commune of Bohicon), Mono (commune of Lokossa) (Fig. 1). These five communes have a subequatorial climate with two rainy seasons (April to June, and September to November) and two dry seasons (July to August, and December to March). The annual rainfall ranges between 1200 and 1300 mm and the humidity is about 75%. All the communes feature rivers, marshes, swamps and lowlands which fostering activities such as market gardening and fish farming.

Additionally, the commune of Djougou, situated in the Donga department, was included in the survey. Djougou exhibits a Sudano-Guinean climate marked by a single rainy season from April to October and a sole dry season from October to April. The annual rainfall in Djougou varies between 900 and 1100 mm, and agriculture stands as the predominant activity in this region.

The selection of these study communes was deliberate, driven by their elevated malaria prevalence and the prevalent vector resistance to pyrethroids [24–26]. Moreover, the extensive use of insecticides in these areas is remarkable, primarily for safeguarding crops against pests.

### Larvae collection, rearing and identification

Larvae and pupae of Anopheles mosquitoes were meticulously gathered from identified positive breeding sites in both central and peripheral areas of each commune, employing a combination of dippers, ladles, pipettes, and larval containers. Subsequently, these specimens were transported to the insectary at the Centre de Recherche Entomologique de Cotonou (CREC) for further examination. Within the controlled environment of the insectary, the larvae and pupae were reared at a temperature of 26 °C ± 1 °C and a relative humidity of 80% until reaching adulthood. Morphological identification exclusively utilized the Coetzee taxonomic key [27], focusing solely on members of the *An. gambiae* s.l. species for subsequent tests.

### WHO susceptibility tube tests

The susceptibility status of *An. gambiae* s.l. populations to pyrethroid insecticides was evaluated through WHO tube tests. Batches of 20–25 unfed female mosquitoes, aged 2–5 days, were exposed to papers treated with deltamethrin 0.05%, permethrin 0.75%, and alpha-cypermethrin 0.05% for a duration of 60 min. Concurrently, control batches of 20–25 mosquitoes were exposed to untreated papers. Throughout the exposure period, the number of mosquitoes knocked down by the insecticide was recorded at 15-min intervals. Following exposure, the mosquitoes were transferred to observation tubes, where they were provided with a 10% sugar solution and maintained at a temperature of 27 °C ± 2 °C with a humidity level of 75% ± 10% for 24 h. The mortality rate was determined 24 h post-exposure [28].

### CDC bottle bioassays

The susceptibility of wild populations of *An. gambiae* s.l. to chlorfenapyr was determined utilizing the CDC bottle bioassay. To conduct this assay, 250 ml glass Wheaton bottles were coated with 1 ml of chlorfenapyr (100 μg/ml), while a bottle coated with 1 ml of acetone served as a control. Batches of 20–25 mosquitoes were introduced into the coated bottles for a 60-min exposure period, during which the number of knocked-down mosquitoes was recorded every 15 min. Subsequent to the exposure, the mosquitoes were gently transferred to observation cups and provided with a 10% sugar solution. Immediate mortality was recorded after 1 h of exposure, and delayed mortalities were subsequently documented at 24, 48, and 72 h post-exposure. Mosquitoes that expired immediately were preserved in RNA later at − 80 °C, whereas those succumbing after 24-, 48- and 72-h post-exposure were preserved in silica gel [29].

### Molecular and biochemical assays

Dead and live mosquitoes from the WHO susceptibility tube tests underwent PCR analysis to ascertain the molecular species within the *An. gambiae* complex [30] and to detect the presence of the *Kdr* L1014F mutation [31].

For biochemical analyses, thirty unexposed female *An. gambiae* s.l., 2 to 5 days old, from each commune underwent biochemical analyses. These analyses aimed to compare the expression levels of detoxification enzymes, including mixed function oxidases, non-specific esterases, and glutathione S-transferases, across diverse field populations of *An. gambiae* s.l. and the reference susceptible strain (*An. gambiae* Kisumu), in accordance with the protocol described by Hemingway et al. [32].

### Description of tested mosquito nets

The three types of new-generation nets include:

- Olyset^®^ Plus: Manufactured by Sumitomo Chemicals, Japan, this polyethylene LLIN incorporates 2% permethrin (800 mg permethrin ai/m^2^), and 1% PBO (400 mg PBO ai/m^2^).
- PermaNet^®^ 3.0: Produced by Vestergaard Frandsen SA, Denmark this LLIN features a polyethylene roof coated with 2.8 g/kg ± 25% deltamethrin and 4.0 g/kg ± 25% PBO. Its polyester sides are coated with 2.8 g/kg ± 25% deltamethrin.
- Interceptor^®^ G2: Manufactured by BASF SE, Ludwigshafen, Germany, this polyester LLIN is coated with a mixture of 200 mg/m^2^ chlorfenapyr and 100 mg/m^2^ alpha-cypermethrin.

In comparison, the standard pyrethroid-only net used as a control is Interceptor^®^, a polyester netting manufactured by BASF SE, Ludwigshafen, Germany, incorporating 200 mg/m^2^ of alpha-cypermethrin.

### WHO cone bioassay

A susceptible laboratory strain (*An. gambiae* Kisumu), and field populations of *An. gambiae* s.l. were utilized to assess the bio-efficacy of new-generation nets according to the WHO cone test protocol [33].

Nets that were brand new, and never used in the community (Olyset^®^ Plus, Interceptor^®^ G2 and PermaNet^®^ 3.0) were tested in this study. Five pieces of net, measuring 30 cm × 30 cm, including a piece from the roof and a piece from each of the four lateral sides were sampled on Olyset^®^ Plus (mixture PBO and Permethrin) and Interceptor^®^ G2 (mixture Chlorfenapyr and alphacypermethrin). For PermaNet^®^ 3.0, four pieces of netting were sampled, two from the roof and two from the sides (one from the length and one from the width), due to the difference in insecticide types between the roof (PBO + Deltamethrin) and the lateral sides (Deltamethrin). All net pieces were individually labelled, securely wrapped in foil, and stored in a refrigerator before testing. During the tests, each net piece had two standard cones affixed using a plastic plate. We introduced five unfed female *An. gambiae* s.l., aged 2 to 5 days, into each cone for a 3-min exposure. Post-exposure, mosquitoes were gently transferred into cups, provided with a 10% sweetened juice, and observed for 24 h at room temperature (27 °C ± 2 °C) with a relative humidity of 75% ± 10%. The number of knocked-down mosquitoes was recorded every 5 min during and one hour after exposure. Mortality rates were determined 24 h post-exposure.

For Olyset^®^ Plus and Interceptor^®^ G2 nets, a total of 50 mosquitoes were tested per net, while 40 were tested for PermaNet^®^ 3.0. This comprehensive testing approach aimed to provide a thorough assessment of the nets' effectiveness against An. gambiae s.l. populations.

### WHO tunnel test

The bio-efficacy assessment of Interceptor^®^ G2 and Interceptor^®^ LLINs also included tunnel tests, with an untreated mosquito net serving as a negative control. In this experiment, unfed female *An. gambiae* s.l., aged 7 days, were released into a tunnel with two square sides (25 cm × 25 cm) and a length of 60 cm. The tunnel was partitioned into two sections: section A, constituting 2/3 of the tunnel where mosquito releases occurred, and section B, encompassing the remaining portion of the tunnel where an immobilized bait (a guinea pig) was placed. At the end of section B, a 25 cm side square cage covered with polyester netting was installed. The netting to be tested was positioned just after section A. The area of the net accessible to mosquitoes measured 400 cm2 (20 cm × 20 cm), featuring nine holes of 1 cm in diameter. In the evening, one hundred unfed female mosquitoes, held without food for a minimum of 6 h before the test, were introduced into the cage through the end of section A. Three separate tunnels were employed for the Interceptor^®^ G2, Interceptor^®^ (positive control), and the untreated net (negative control). Following 12 h of exposure, mosquitoes were carefully removed from each section of the tunnel in the early morning using a mechanical aspirator and placed in veiled and labeled cups. Mosquitoes in these cups were provided with a 10% sugar solution and observed for 72 h to determine the delayed mortality. The number of live, dead, unfed or blood-fed mosquitoes in each section of the tunnel was recorded to determine the entry, mortality, and blood-feeding inhibition rates. The tests were conducted in total darkness overnight, maintaining a constant temperature of 27 ± 2 °C and a relative humidity of 75% ± 10% [34].

### Data analysis

The mortality rates observed 24 h after exposure to various insecticides were interpreted in accordance with WHO criteria [28]:

- Mortality rate between 98 and 100%: Susceptible mosquito population
- Mortality rate ≥ 90% and < 98%: Possible resistance in the mosquito population
- Mortality rate < 90%: Insecticide-resistant mosquito population.

The allelic frequencies of the *kdr* L1014F mutation were determined by the following formula: F = (2nRR + nRS) / (2(nRR + nRS + nSS)).

n = number of genotypes, RR: homozygous resistant, RS: Heterozygous, SS: homozygous susceptible.

The exact binomial test was used to calculate confidence intervals for mortality rates and allelic frequencies of *kdr* L1014F mutations. To assess the resistance activity of metabolic enzymes, their expression level was compared between field populations of *An. gambiae* s.l. and the laboratory susceptible strain, *An. gambiae* (Kisumu strain). GraphPad Prism8 software was used to draw the graphs and calculate the p-values.

The Mann–Whitney U test enabled comparison of the activity of enzymes, between the field mosquito populations and the laboratory susceptible strain (*An. gambiae* Kisumu). Statistical analyses were conducted using R 3.3.2 software [35].

Data on the bioefficacy of LLINs tested with the laboratory susceptible strain *An. gambiae* (Kisumu strain) was analyzed according to WHO criteria:

- Minimal efficacy: KD60 ≥ 75% or mortality rate ≥ 50%.
- Optimal efficacy: knock down rate at 60 min (KD60) ≥ 95% or mortality rate ≥ 80%.

Furthermore, mortality rates displayed by LLINs using wild populations of *An. gambiae* s.l. was also presented.

The indicators evaluated through the tunnel tests are as follows:

- Blood-feeding rate (%) = (A/B) × 100, where A is the number of blood-fed mosquitoes collected in the tunnel, and B is the total number of mosquitoes exposed to the insecticide-incorporated net.
- Blood-feeding inhibition rate (%) = ((C − D))/C × 100, where C and D are the blood feeding rates obtained with the untreated, and insecticide-treated nets, respectively.
- Immediate mortality (%) = (E/F) × 100, where E is the number of dead mosquitoes collected in the tunnel just after the 12-h-exposure time, and F is the total number of mosquitoes exposed to the insecticide-incorporated net.
- 24-h mortality (%) = (G/H) × 100, where G is the number of dead mosquitoes 24 h post-exposure, and H, the total number of mosquitoes exposed to the insecticide-treated net
- 72-h mortality (%) = (I/J) × 100, where I is the number of dead mosquitoes within 72 h, and J, the total number of mosquitoes exposed to the insecticide-treated net.

## Results

### Susceptibility of *An. gambiae* s.l. to pyrethroids (WHO susceptibility tube tests)

**Fig. 2:**
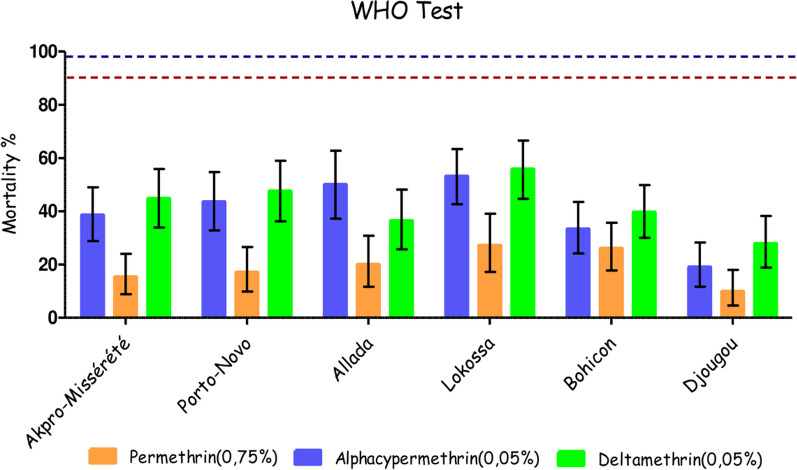
Mortality rate of *An. gambiae* s.l. populations after 60 min exposure to alpha-cypermethrin (0.05%), permethrin (0.75%) and deltamethrin (0.05%) after 24 h observation

Overall, the populations of *An. gambiae* s.l. from Akpro-Missérété, Porto-Novo, Bohicon, Lokossa, Allada, and Djougou showed high pyrethroid resistance. The mortality rates were consistently below 60% for all pyrethroid insecticides tested, regardless of the commune. Specifically, the rates ranged from 9.89% (95% CI: 4.62–17.95) to 27.14% (95% CI: 17.20–39.10) for permethrin, 18.95% (95% CI: 11.63–28.28) to 53.13% (95% CI: 42.66–63.39) for alphacypermethrin, and 27.78% (95% CI: 18.95–38.22) to 55.81% (95% CI: 44.70–66.52) for deltamethrin (Fig. 2).

### Frequency of kdr L1014F mutation in *An. gambiae* s.l.

Across all studied populations of *An. gambiae* s.l. (Akpro-Missérété, Porto-Novo, Bohicon, Lokossa, Allada, and Djougou), the prevalence of the *kdr* L1014F mutation was high. The average frequency was approximately 86% (95% CI: 83–88) across the entire study area. Akpro-Missérété exhibited the lowest rate at 83% (95% CI: 77–88), while Allada displayed the highest at 89% (95% CI: 81–94). No significant differences were observed in *kdr* frequencies among the study sites or between the two species of the *An. gambiae* s.l. complex, with *An. gambiae* at 87% (95% CI: 84–90) and *An. coluzzii* at 84% (95% CI: 80–88) (Table 1). The mean difference of the *kdr* L1014F frequency between the two molecular species was 3% (95% CI: 2.8–3.5).

**Table 1:** Frequency of Kdr L1014F mutations in *An. gambiae* s.l. populations

Communes/species	No. tested	Genotypes			*Freq. 1014F (%)*	CI
		*1014F*	*1014F*	*1014L*		
		*1014F*	*1014L*	*1014L*		
Akpro-Missérété	99	73	19	7	83	[77–88]
* An. coluzzii*	63	45	14	4	83	[75–89]
* An. gambiae*	36	28	5	3	85	[74–92]
Porto-Novo	50	39	7	4	85	[76–91]
* An. coluzzii*	50	39	7	4	85	[76–91]
Bohicon	50	35	15	0	85	[76–91]
* An.coluzzii*	26	19	7	0	87	[74–94]
* An. gambiae*	24	16	8	0	83	[70–93]
Lokossa	99	80	13	6	87	[82–92]
* An. coluzzii*	50	37	8	5	82	[73–89]
* An. gambiae*	49	43	5	1	93	[86–97]
Allada	50	40	9	1	89	[81–94]
* An. coluzzii*	12	8	4	0	83	[63–95]
* An. gambiae*	38	32	5	1	91	[82–96]
Djougou	49	38	7	4	85	[76–91]
* An. coluzzii*	7	7	0	0	100	[77–100]
* An. gambiae*	42	31	7	4	82	[72–90]
All area	397	305	70	22	86	[83–88]
* An. coluzzii*	208	155	40	13	84	[80–88]
* An. gambiae*	189	150	30	9	87	[84–90]

*An Anopheles*, *Freq* frequency, *No* number, %: Percentage, CI: Confidence Interval

### Enzymatic activities in *An. gambiae* s.l.

**Fig. 3:**
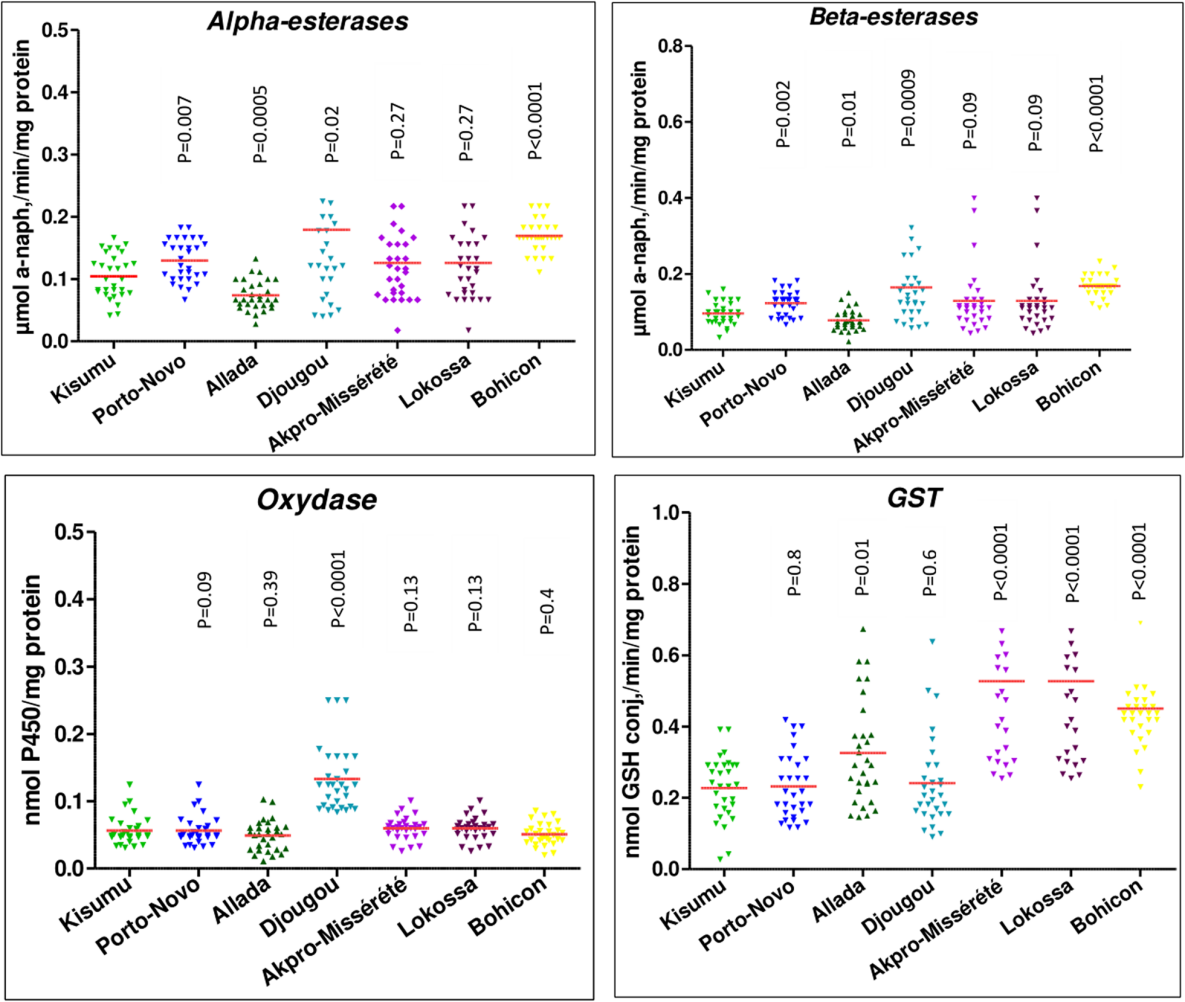
Enzymatic activity of different populations of *An. gambiae* s.l. p < 0.05 indicates a significant difference of the expression level of an enzyme between a field-collected mosquito population and the Kisumu susceptible strain

Enzyme activity was assessed by comparing field populations of *An. gambiae* s.l. with the susceptible laboratory strain Kisumu. The findings revealed an overexpression of glutathione-s-transferases (GST) in Allada, Akpro-Missérété, Lokossa, and Bohicon. Additionally, significantly elevated esterase activity was noted in Djougou, Porto-Novo and Bohicon as compared to the Kisumu susceptible strain. The overproduction of oxidases (MFOs) was specifically observed in Djougou (Fig. 3).

### Susceptibility of *An. gambiae* s.l. to chlorfenapyr (CDC bottle bioassay)

**Fig. 4:**
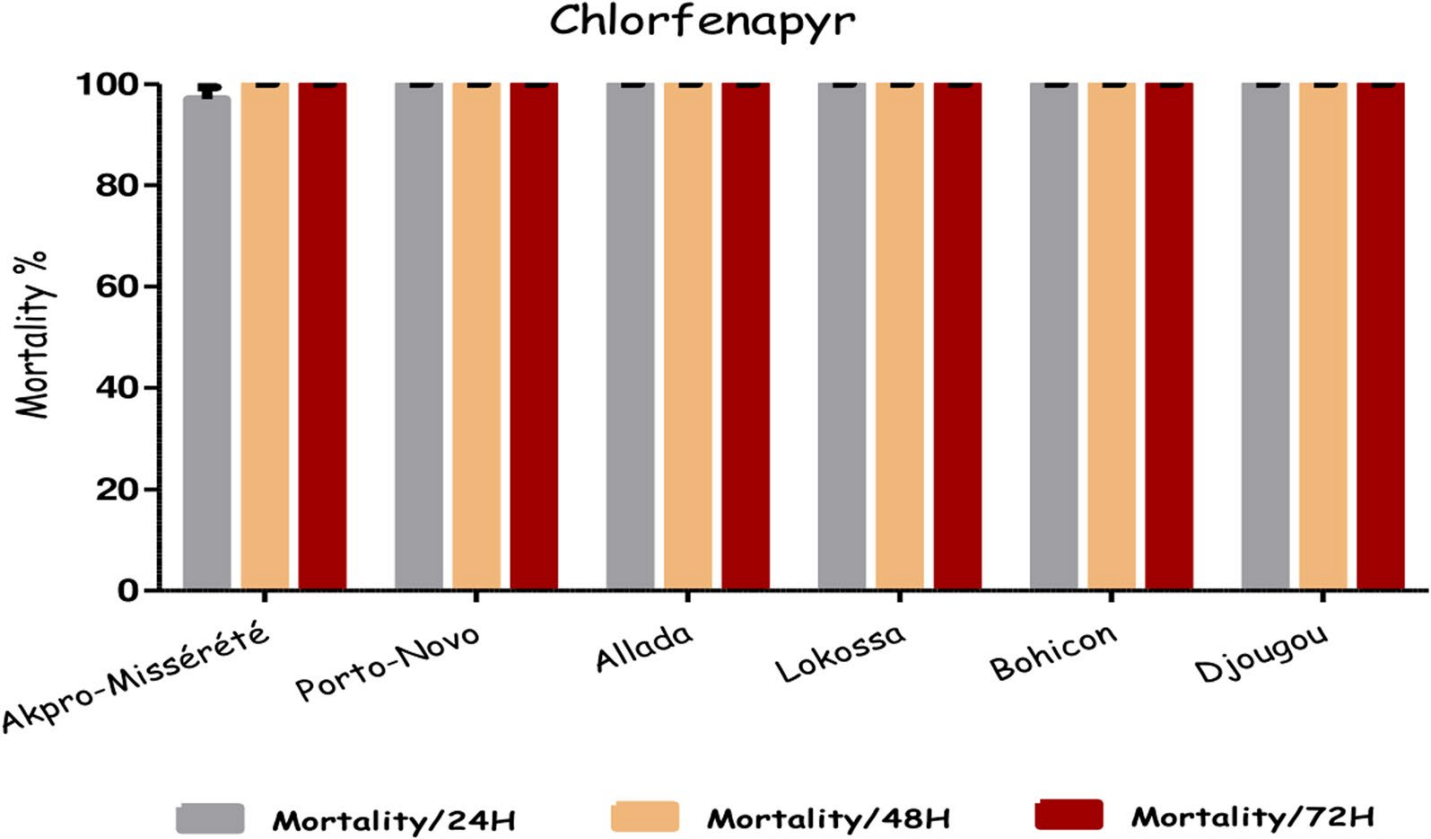
Sensitivity of *An. gambiae* s.l. populations to chlorfenapyr

Figure 4 depicts the mortality rates of six field populations of *An. gambiae* s.l. exposed to chlorfenapyr. All mosquito populations exhibited complete sensitivity to chlorfenapyr, with 100% mortality observed between 24- and 48-h post-exposure. No significant differences were noted in mortality rates at 24, 48 and 72 h after exposure to the insecticides. Specifically, the rates were 97% (95% CI: 91.48–99.38) in Akpro-Missérété, and 100% in Porto-Novo (95% CI: 96.45–100), Allada (95% CI: 96.23–100%), Lokossa (95% CI: 96.61–100), Bohicon (95% CI: 96.38–100), and Djougou (95% CI: 96.82–100) at 24 h after exposure, and remained at 100% for all mosquito populations at 48 h and 72 h post-exposure.

### WHO cone bioassay results

**Fig. 5:**
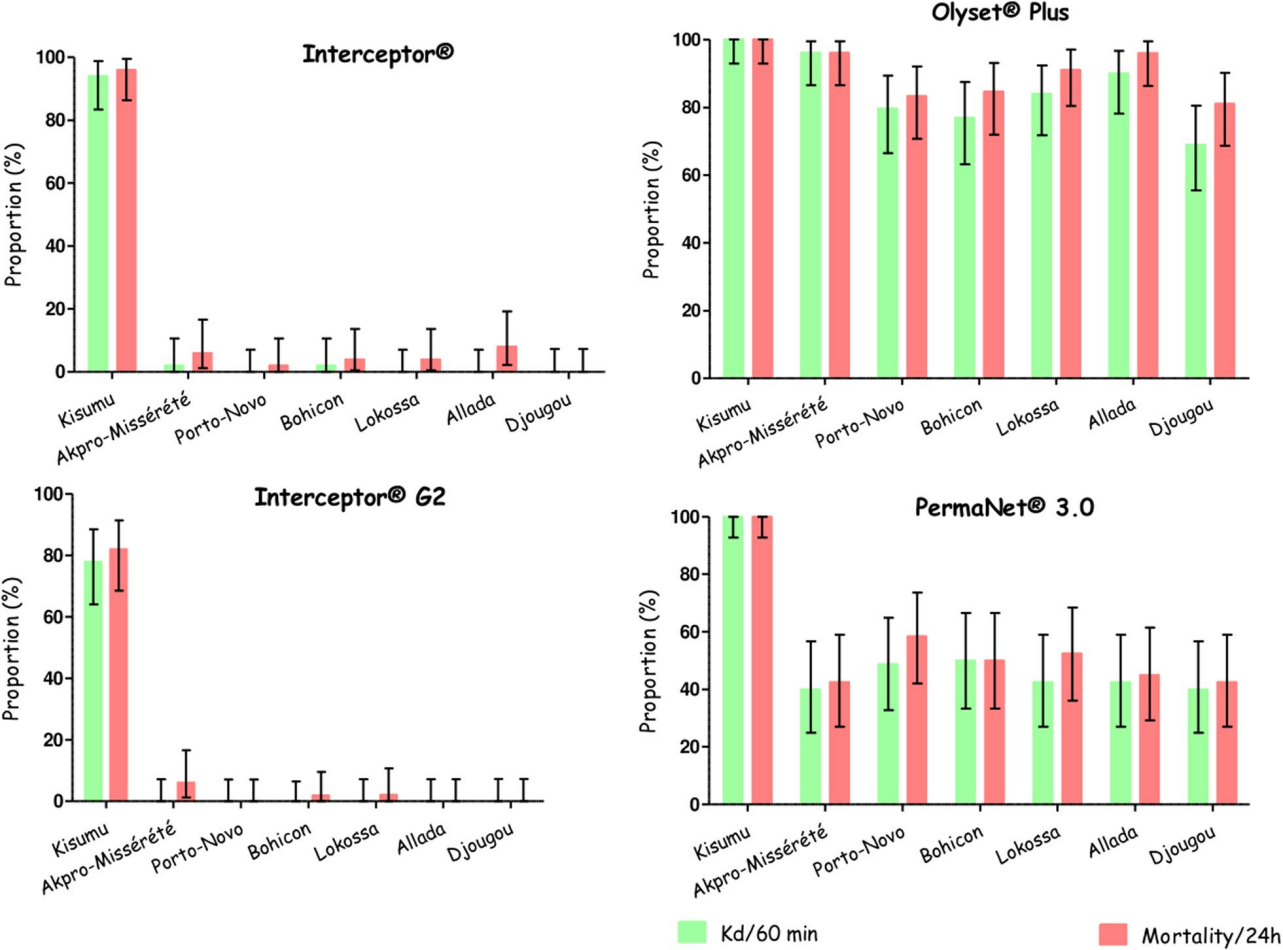
Bioefficacy of new-generation nets

Not only did Olyset^®^ Plus and PermaNet^®^ 3.0 nets exhibit optimal efficacy (mortality ≥ 80%) with the laboratory-susceptible strain (*An. gambiae* Kisumu), but they also demonstrated significantly higher mortality rates with field populations of *An. gambiae* s.l. compared to the Interceptor^®^ net. Similarly, Interceptor^®^ 96% (95% CI: 86–100) and Interceptor^®^ G2 82% (95% CI: 68.56–91.42) nets displayed optimal efficacy with the susceptible strain, with a difference of 14% (95% CI: 0.04–27.95). However, the highest mortality induced by LLIN Interceptor^®^ on the field population of *An. gambiae* s.l. after 24 h of exposure was 8% (95% CI: 2–19) in Allada, and 6% (95% CI: 1.25–16.55) in Akpro-Missérété with LLIN Interceptor^®^ G2. For Olyset^®^ Plus, the mortality rates were 100% (95% CI: 92.89–100) with the Kisumu strain, 96.08% (95% CI: 86.54–99.52) in Akpro-Missérété, 83.33% (95% CI: 70.71–92.08) in Porto-Novo, 84.62% (95% CI: 71.92–93.12) in Bohicon, 91.07% (95% CI: 80.38–97.04) in Lokossa, 96% (95% CI: 86.29–99.51) in Allada, and 81.03% (95% CI: 68.59–90.13) in Djougou. With PermaNet^®^ 3.0, the mortality rates were 100% (95% CI: 92.89–100) with Kisumu, 42.50% (95% CI: 27.04–59.11) with Akpro-Missérété, 58.54% (95% CI: 42.11–73.68) with Porto-Novo, 50% (95% CI: 33.38–66.62) in Bohicon, 52.50% (95% CI: 36.13–68.49) in Lokossa, 45% (95% CI: 29.26–61.51) in Allada, and 42.5% (95% CI: 27.04–59.11) in Djougou. No significant difference was observed between the mortality rates of the six *An. gambiae* s.l. populations exposed to Olyset^®^ Plus and PermaNet^®^ 3.0 nets, respectively. (Fig. 5).

### WHO tunnel test results

**Fig. 6:**
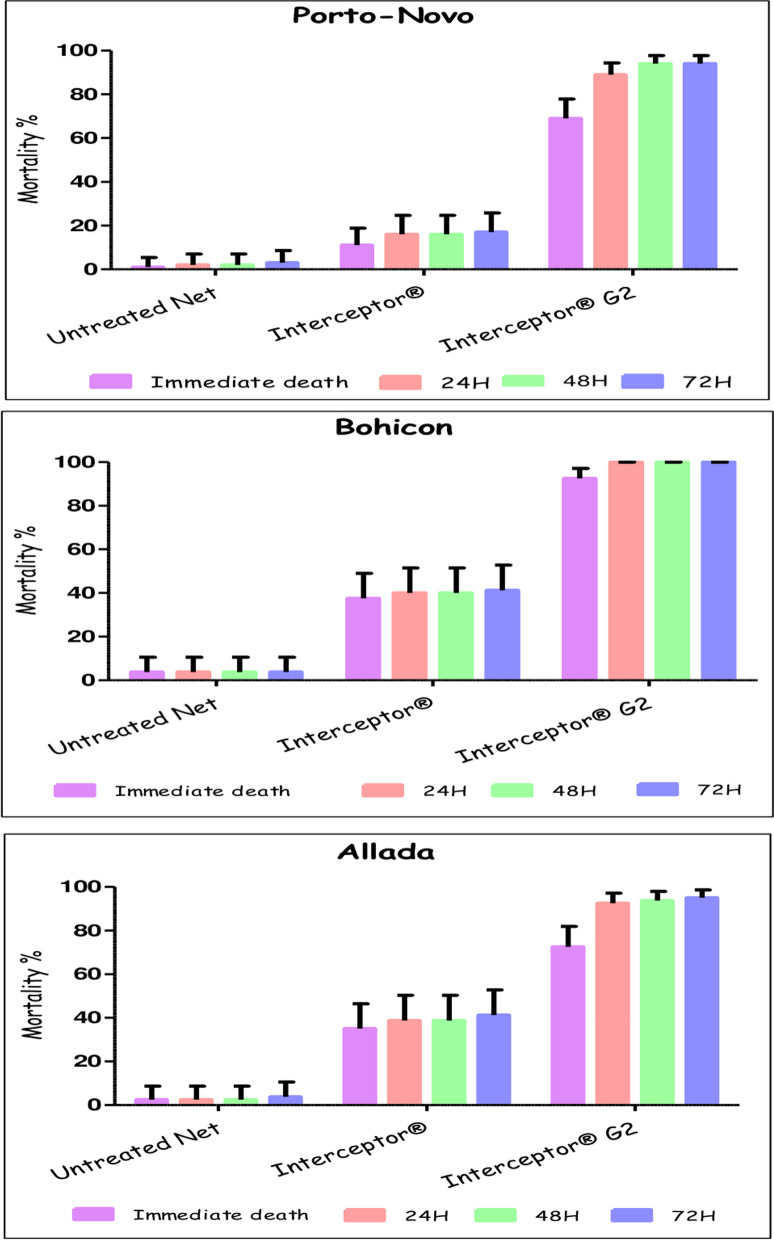
Mortality of *An. gambiae* s.l. populations after tunnel test

In the tunnel test, Interceptor^®^ G2 outperformed Interceptor^®^. Immediate mortality rates observed with Interceptor^®^ G2 were 69% (95% CI: 58.97–77.87), 92.5% (95% CI: 84.39–97.2), and 72.5% (95% CI: 61.38–81.9) in Porto-Novo, Bohicon, and Allada, respectively. In contrast, for Interceptor^®^, the rates were 11% (95% CI: 5.62–18.83), 37.5% (95% CI: 26.92–49.04), and 35% (95% CI: 24.67–46.48) in the same locations. A substantial difference in mortality rates of all *An. gambiae* s.l. populations was observed between these two nets. Twenty-four hours after exposure, mortality rates were significantly higher with Interceptor^®^ G2, reaching 89% (95% CI: 81.17–94.38), 100% (95% CI: 95.49–100), and 92.5% (95% CI: 84.39–97.20) in Porto-Novo, Bohicon, and Allada, respectively. In contrast, Interceptor^®^ showed immediate mortality rates that did not differ significantly from those observed after 24 h 16% (95% CI: 9.43–24.68), 40% (95% CI: 29.20–51.56), and 38.75% (95% CI: 28.06–50.30), 48 h, and 72 h (Figs. 5, 6).

The blood-feeding rate was 6% (95% CI: 2.23–12.6) for Interceptor^®^ G2, 22% (95% CI: 14.33–31.39) for Interceptor^®^ and 55% (95% CI: 44.73–64.97) for the untreated net in Porto-Novo. In Bohicon, the rates were 6.25% (95% CI: 2.06–13.99) for Interceptor^®^ G2, 31.25% (95% CI: 21. 35–42.59) for Interceptor^®^ and 65% (95% CI: 53.52–75.33) for the untreated net. In Allada, the rates were 0% (95% CI: 0–4.51) for Interceptor^®^ G2, 22.5% (95% CI: 13.91–33.21) for Interceptor^®^ and 55% (95% CI: 43.47–66.15) for the untreated net. A significant difference was observed between blood-feeding rates observed with Interceptor^®^ G2 and Interceptor^®^.

In terms of blood-feeding inhibition rates, Interceptor^®^ G2 demonstrated higher rates than Interceptor^®^. The inhibition rates were 89.09%, 90.38% and 100% for Interceptor^®^ G2 compared to 60%, 51.92% and 59.09% for Interceptor^®^ in Porto-Novo, Bohicon and Allada respectively (Table 2).

**Table 2:** Results of tunnel tests

Communes	Mosquito net	Number tested	Immediate mortality % (CI)	OR (CI)	p-value	Entry rate %	OR (CI)	p-value	Blood feeding rate % (CI)	Blood feeding inhibition (%)
						(CI)				
Porto-Novo	Untreated Net	100	1 [0.03–5.45]	1 [NA]	0.002	53 [42.76–63.06]	1 [NA]	4.55	55 [44.73–64.97]	60
	Interceptor^®^	100	11 [5.62–18.83]	12.12 [1.70–530.54]		33 [23.92–43.12]	0.43 [0.23–0.80]		22 [14.33–31.39]	
	Untreated Net	100	1 [0.03–5.45]	1 [NA]	0.00	53 [42.76–63.06]	1 [NA]	2.88	55 [44.73–64.97]	89.09
	Interceptor^®^ G2	100	69 [58.97–77.87]	214.06 [34.19–8389.62]		16 [9.43–24.68]	0.17 [0.08–0.34]		6 [2.23–12.6]	
Bohicon	Untreated Net	80	3.75 [0.78–10.57]	1 [NA]	4.56	56.25 [44.7–67.32]	1 [NA]	2.82	65 [53.52–75.33]	51.92
	Interceptor^®^	80	37.5 [26.92–49.04]	15.16 [4.36–81.70]		38.75 [28.06–50.3]	0.49 [0.24–0.96]		31.25 [21.35–42.59]	
	Untreated Net	80	3.75 [0.78–10.57]	1 [NA]	0.00	56.25 [44.7–67.32]	1 [NA]	1.16	65 [53.52–75.33]	90.38
	Interceptor^®^ G2	80	92.5 [84.39–97.2]	286.04 [67.78–1867.59]		16.25 [8.95–26.18]	0.15 [0.06–0.33]		6.25 [2.06–13.99]	
Allada	Untreated Net	80	2.5 [0.3–8.74]	1 [NA]	3.65	60 [48.44–70.8]	1 [NA]	1.70	55 [43.47–66.15]	59.09
	Interceptor^®^	80	35 [24.67–46.48]	20.66 [4.86–186.14]		26.25 [17.04–37.29]	0.23 [0.11–0.48]		22.5 [13.91–33.21]	
	Untreated Net	80	2.5 [0.3–8.74]	1 [NA]	0.00	60 [48.44–70.8]	1 [NA]	4.09	55 [43.47–66.15]	100
	Interceptor^®^ G2	80	72.5 [61.38–81.9]	98.94 [23.17–917.26]		7.5 [2.8–15.61]	0.05 [0.01–0.14]		0 [0–4.51]	

*CI* confidence interval, *OR* odds ratio, %: Percentage

## Discussion

The current study is a phase 1 trial assessing the effectiveness of PermaNet^®^ 3.0, Olyset^®^ Plus, and Interceptor^®^ G2, three new generation LLINs on field populations of *An. gambiae* s.l. from Benin.

Insecticide susceptibility tests revealed that all field populations of *An. gambiae* s.l. were resistant to pyrethroids but fully susceptible to chlorfenapyr, aligning with recent findings in several African countries [36]. The widespread pyrethroid resistance, a common issue in Benin, poses a challenge to mosquito control efforts relying solely on pyrethroid-based tools [23, 37, 38]. However, the observed susceptibility to chlorfenapyr suggests its potential as a valuable alternative due to its unique mode of action, disrupting ATP formation in insect mitochondria [20].

The Kdr L1014F mutation, a key contributor to pyrethroid resistance, was prevalent in both *An. gambiae* and *An. coluzzii*, across all study areas, nearing fixation [39]. This could be attributed to the extensive use of pyrethroids for various purposes, exerting high selective pressure. Additionally, Djougou exhibited overexpression of MFOs, indicating a potential fitness cost associated with resistance induced by oxidases [40, 41]. Elevated GSTs and esterases in some study areas further highlight concerns as these enzymes contribute to the expansion of resistance mechanisms.

Given the widespread pyrethroid resistance in malaria vectors, the effectiveness of pyrethroid-only LLINs in protecting against mosquito bites is questionable. Calls to design new-generation nets date back to 2007 [38], with trials suggesting that PBO or next-gen insecticides like chlorfenapyr may offer improved control of resistant vectors [42, 43]. PBO has shown potential to restore susceptibility to pyrethroids, while chlorfenapyr induces full susceptibility in pyrethroid-resistant malaria vectors.

The WHO cone tests performed with the *An. gambiae* (Kisumu strain), indicated optimal efficacy for all three new-generation nets and the pyrethroid-only net (Interceptor^®^), with 24-h mortality rates greater than 80%. However, when tested with wild populations of *An. gambiae* s.l., Olyset^®^ Plus exhibited the highest efficacy, followed by PermaNet^®^ 3.0 and Interceptor^®^ G2. Similar results were reported by Ngofur et al. [17] in experimental huts and Allossogbé et al. [16] with PBO LLINs on multidrug-resistant populations of *An. gambiae* s.l. in Benin. However, the 72-h mortality rates for Interceptor^®^ G2, incorporating chlorfenapyr (a slow-acting insecticide), were not explored, a limitation of this study.

Tunnel test data, assessing immediate mortality, blood-feeding inhibition, and mosquito entry rates, favored Interceptor^®^ G2 LLINs over both Interceptor^®^ LLIN and the untreated net. However, WHO cone tests performed with wild populations of *An. gambiae* s.l. indicated lower mortalities for Interceptor^®^ G2, similar to Interceptor^®^ LLIN, possibly due to the slow-acting nature of chlorfenapyr. The same observation was previously made by Oxborough et al. [44] who ended up concluding that tunnel tests remain the most reliable tests for evaluating the efficacy of new-generation LLINs. According to Oxborough et al. [44] and Kibondo et al. [45], the mode of action of chlorfenapyr (a non-neurotoxic insecticide) is compatible with the circadian activity rhythm of *Anopheles* mosquitoes, as they are quite active overnight but not during the day. This could justify the high mortality rates observed in the tunnel tests during which mosquitoes were exposed overnight to pieces of Interceptor^®^ G2 LLINs. In fact, during the tunnel tests, the mosquitoes attempt to pass through the torn nets to seek the host, which increases the net-vector contact and leads to the disruptive action of chlorfenapyr on the respiratory tract of the mosquitoes.

This study establishes the efficacy of chlorfenapyr-pyrethroid and PBO-pyrethroid LLINs on pyrethroid-resistant mosquito populations in phase 1. While community efficacy has been demonstrated through randomized controlled trials [21, 22, 46–48], deployment considerations should account for local vector resistance levels. Alternating Interceptor^®^ G2 LLINs and PBO LLINs might be a strategy to manage insecticide resistance.

The lack of PBO-pyrethroid susceptibility tests and bioassays with washed LLINs is acknowledged as a limitation, but the study still provides valuable insights into the response of pyrethroid-resistant *An. gambiae* s.l., to new generation LLINs incorporating insecticides with different modes of action, guiding Benin's malaria vector control policy.

## Conclusion

The study underscores the superior efficacy of LLINs incorporating piperonyl butoxide (PBO), such as Olyset^®^ Plus and PermaNet^®^ 3.0, and those with dual-active chlorfenapyr, like Interceptor^®^ G2, against pyrethroid-resistant *An. gambiae* s.l. populations when compared to conventional LLINs such as Interceptor^®^. Remarkably, Olyset^®^ Plus exhibited the highest mortality rates in cone tests, showcasing its potency in controlling pyrethroid-resistant mosquitoes. Interceptor^®^ G2, besides its high mortality rates in tunnel tests, demonstrated superior protection by significantly inhibiting blood feeding. These innovative vector control tools present a promising option for more effective management of pyrethroid-resistant malaria vectors in Benin. However, for optimal efficacy, the distribution of these new LLINs should be tailored to the specific resistance mechanisms prevalent in each agro-ecological zone. Priority distribution of PBO LLINs is recommended in areas where metabolic resistance mechanisms are dominant, with the goal of enhancing protection against mosquito bites for local populations. This targeted approach will contribute to maximizing the impact of these novel tools in diverse malaria-endemic regions.

## Data Availability

The datasets that were analyzed in this study are available from the corresponding author and the lead author.

## Abbreviations

CREC: Centre de Recherche Entomologique de Cotonou
LLINs: Long-Lasting Insecticide-treated mosquito Nets
PBO: Piperonyl butoxide
ATP: Adenosine triphosphate
*An. gambiae*: *Anopheles gambiae*
GST: Glutathione-S-transferases
MFOs: Mixed function oxygenase
WHO: World Health Organisation
